# Proceedings of Societies

**Published:** 1861-01

**Authors:** 


### CHICAGO ACADEMY OF MEDICAL SCIENCES.

The Third Annual Meeting of the Academy was held at its rooms, January 4th, 1861.

The following named fellows were elected officers for the ensuing year:

President—James Bloodgood, M. D.

Vice President—Thomas Bevan, M. D.

Recording Secretary—E. L. Holmes, M. D.

Corresponding Secretary—J. McAllister, M. D.

Treasurer—W. S. Denniston, M. D.

Trustee—A. Fisher, M. D.

Committee on Medical Ethics—R. C. Hamill, M. D.; N. S. Davis, M. D.; E. Powell, M. D.

Committee on Medical Education—W. H. Byford, M. D.; J. W. Freer, M. D.; Thomas Bevan, M. D.

Committee on Admissions—S. Wickersham, M. D.; H. W. Jones, M. D.; S. C. Blake, M. D.

### ABSTRACT OF THE PROCEEDINGS OF THE AURORA CITY MEDICAL ASSOCIATION.

Aurora, Ill., *January 7th*, 1861.

The Aurora City Medical Association met at Dr. Young's office.

Present—The Vice-President, Dr. L. A. Young, in the chair; Drs. Allaire, Angell, Hard and Young.

Minutes of last meeting read and approved.

It being the annual meeting, on motion of Dr. Young, the

Association proceeded to the election of officers for the ensuing year, after which the following gentlemen were declared duly elected:

President—L. H. Angell, M. D.

Vice-President—Abner Hard, M. D.

Treasurer—P. A. Allaire, M. D.

Secretary—D. W. Young, M. D.

On motion of Dr. Allaire, Dr. Young was appointed a committee of one to prepare and forward to the Hon. Edward R. Allen, our Representative in the Legislature, a copy of the petition presented to and recommended by the Illinois State Medical Society, at its tenth annual meeting, petitioning the Legislature to pass a registration law for the State of Illinois.

On motion of Dr. Young, Dr. Allaire was appointed a special committee to wait upon and inform the Common Council that their ordinance requiring the registration of deaths and all interments occurring within the city limits, was being violated to a certain extent, especially at the Catholic Cemetery, and requesting them to see that the ordinance should be enforced in every case in the future.

On motion of Dr. Young, Dr. Howell, the retiring President, being unable to attend this meeting, was granted further time to deliver his valedictory address.

The President appointed Dr. Allaire to read an address at the next meeting. The President announced Scarlet Fever as the subject for discussion at the next meeting.

On motion of Dr. Hard, the Secretary was instructed to forward an abstract of the proceedings of this meeting to the *Chicago Medical Journal* for publication.

On motion of Dr. Young, the Association adjourned to meet at Dr. Allaire's office, on Monday evening, January 21st, 1861.

D. W. YOUNG, M. D., SECRETARY.

### PROCEEDINGS OF THE SEMI-ANNUAL MEETING OF THE ROCK RIVER UNION MEDICAL SOCIETY FOR 1861.

The Society convened at Dixon, Jan. 16th. The President, Dr. A. G. Porter, being absent, the meeting was organized, on motion of Dr. G. W. Phillips, by choosing Dr. Moses M. Roger, of Sterling, as President *pro. tem.*

Proceedings of last meeting read and approved.

Drs. J. F. Marsh, and P. J. Wardner, of Grand de Tour, being proposed as members, were, on motion, admitted members of the Society.

Report of Committee on Surgery being called for, Dr. G. W. Phillips, as a member of the committee, read an essay, entitled: "The line of demarcation, and of separation—the course of its formation—and its practical significance."

On motion of Dr. N. W. Abbott, the Report was accepted and ordered to be placed on file, with the papers of the Society.

Drs. Abbott, Wardner, and W. Wynn, made some observations upon the practical points contained in the essay.

A discussion here sprung up as to the possibility of a limb, as a toe or finger, after having been completely severed from its connection, again being united.

Dr. Wardner mentioned a case, which came under his observation, of the severing of the foot, through the middle, by incised wound, so that a portion, or isthmus of skin, not an half an inch wide, remained; the wound, after being properly dressed, united again firmly, so as to give an useful limb.

Dr. Roger mentioned a case, for which he vouched, but did not see, of the complete severing of a finger, which, being immediately placed back to its place, and firmly retained, united strongly, and gave an useful finger.

Dr. Abbott, from committee on "Best means of preparing

Medicines for convenient administration to young children," made remarks upon the subject. He had found that common salt, taken in the mouth, held, and then swallowed, both before and after taking a dose of quinine, effectually destroyed the taste of the medicine.

On motion, adjourned until after tea.

---

*Evening Session.*—On motion, the question of printing the fee-bill was laid over until the annual meeting in June.

On motion of Dr. Phillips, the President appointed three delegates, consisting of Drs. N. W. Abbott and G. W. Phillips, of Dixon, and Dr. A. M. Freas, of Milledgeville, Carroll Co., to attend the American Medical Association, to be held in Chicago next June.

On motion, Drs. H. C. Donaldson, of Como, Moses M. Roger, of Sterling, and Dewitt C. Warney, of Dixon, were chosen delegates to the State Medical Society.

On motion, the committees that have not reported, be continued, and report at the annual meeting.

On motion, Dr. A. W. Benton was admitted a member of the Society.

On motion of Dr. Abbott, the Secretary was instructed to furnish a copy of proceedings for publication in the Chicago *Med. Journal*, and in the *Republican* and *Telegraph*, of Dixon.

On motion of Dr. Phillips, Sterling was selected as the place for holding the annual meeting.

On motion, adjourned.

G. W. PHILLIPS, M. D., SEC'Y.

---

*Unity of the Human Race.*—The veteran Dr. Samuel A. Cartwright, contributes to De Bow's *Review* a characteristic article, wherein he argues from the Hebrew Bible that the unity of the human race is theologically disproved. We opine, however, that opinions will still continue to differ on the point.

